# The development of the Compassion Satisfaction and Compassion Fatigue scale

**DOI:** 10.3389/fpubh.2024.1406467

**Published:** 2024-12-05

**Authors:** Júlia Halamová, Martin Kanovský, Katarina Krizova, Bronislava Šoková, Martina Baránková, Charles Figley

**Affiliations:** ^1^Institute of Applied Psychology, Faculty of Social and Economic Sciences, Comenius University in Bratislava, Bratislava, Slovakia; ^2^Institute of Social Anthropology, Faculty of Social and Economic Sciences, Comenius University in Bratislava, Bratislava, Slovakia; ^3^Traumatology Institute, Tulane University, New Orleans, LA, United States

**Keywords:** compassion fatigue, compassion satisfaction, helping professionals, psychometrics, self-testing

## Abstract

**Background:**

There is a high probability of compassion fatigue occurring in helping professionals who work with traumatized clients or patients. Several instruments exist for measuring compassion fatigue, but all of them have methodological flaws. The original Compassion Satisfaction/Fatigue Self-Test for Helpers is time-consuming and its psychometric properties, including factor structure, have not been supported in the research.

**Methods:**

Therefore, the goal of this study was to apply a Mokken scale analysis for polytomous items to shorten the Compassion Satisfaction/Fatigue Self-Test for Helpers and improve its psychometric properties. In addition, we wanted to create norms for the helping professional population. The research sample consisted of 2,320 participants from various helping professions.

**Results:**

To improve scalability, most of the scale items were removed. The resulting item scalability coefficients ranged from 0.349 to 0.655 and Molenaar–Sijtsma reliability coefficient ranged between 0.75 and 0.87. The final revised and shortened Compassion Satisfaction and Compassion Fatigue scale (CSCFS) consisted of 5 items for the Compassion Satisfaction—Personal Integrity and Happiness subscale, 5 items for the Compassion Satisfaction—Work Competence and Happiness subscale, 9 items for the Compassion Fatigue—Secondary Traumatic Stress subscale, and 7 for the Compassion Fatigue—Burnout subscale. The newly revised subscales have good reliability coefficients.

**Conclusion:**

The CSCFS appears to be a valid and reliable instrument for assessing compassion satisfaction and compassion fatigue among helping professionals. More research is required to support its factor structure in a range of settings. We recommend testing usability across different helping professions and cultures.

## Compassion fatigue and compassion satisfaction

The first person to use the term compassion fatigue was Joinson (54), who noted that nurses who care about their patients may also suffer because they internalize various kinds of stress from their patients. This was later named as the “cost of care” (1). The most well-known person associated with the term compassion fatigue is Figley (2), who suggests that being compassionate has negative consequences for the individual. When empathizing with the suffering of others, the individual often end up suffering themselves. Therefore, compassion fatigue can be defined as a state of emotional and physical exhaustion that leads to a reduced capacity for empathy or compassion and to a reduced capacity to bear the suffering of others (2).

According to Stamm (64), there are two aspects to the compassion experienced by professionals: positive (compassionate satisfaction) and negative (compassion fatigue). Compassion fatigue consists of two parts: burnout and secondary traumatic stress. Burnout is related to workplace stressors and associated with feelings of hopelessness and difficulty coping with work; while secondary traumatic stress is linked to exposure to traumatic stressful events, resulting in fear, sleeping problems, intrusive thoughts, or avoidance.

Compassion fatigue is usually associated with various symptoms (60) resulting from care provision and trauma exposure that are either related to first-hand (primary) trauma or the provision of care to those who have experienced trauma (secondary trauma). Figley (2) describes seven areas affected by compassion fatigue: cognitive, emotional, behavioral, spiritual, personal relations, somatic, and work performance. In the literature several terms are used to describe the negative effects of helping, such as compassion fatigue, secondary traumatic stress, second victim traumatization, client-related burnout, and vicarious trauma. Although there has been some discussion on whether these all these terms refer to the same construct (e.g., 62, 63), so far there is no evidence of differences between these concepts (64). In contrast to compassion fatigue, many helping professionals also have positive experiences of helping, known as compassion satisfaction (64). The term compassion satisfaction refers to the pleasure and satisfaction derived from being able to help others and being committed to and effective in their work (3).

Compassion fatigue is not usually triggered by a single encounter with trauma, but by constant, repetitive exposure to trauma. The costs are huge as it increases the likelihood of mistakes being made and reduces work performance, while leaving helping professionals vulnerable to becoming cold, cynical, robotic, demotivated, and exhausted and, more worryingly, it affects their ability to provide good care (4). Compassion fatigue can impair the ability of helping professionals to provide help and could result in unprofessional decisions, misdiagnosis, mistreatment, malpractice, and even client abuse (5). Therefore, early detection of the signs of compassion fatigue could provide helping professionals with time to learn new coping skills and techniques to prevent the full onset of compassion fatigue (4). This is even more important in settings where insufficient care can have enormous consequences for the physical or mental health of patients or clients, such as in healthcare settings (6). Based on the recent systematic review and meta-analysis of a total of 71 studies by Cavanagh et al. (7) compassion fatigue is distinct from “burnout” and represents a form of psychological distress that can be insidious, affecting all health professions and potentially impairing their ability to deliver care. Professionals who experience high levels of compassion fatigue also often report having various psychopathological symptoms, including substance use, depression, anxiety, and suicidal ideation (55).

These conditions not only undermine the mental health and well-being of healthcare providers but also adversely impact the quality of care they deliver. High levels of these stressors are linked to increased medical errors, lower patient satisfaction, and higher turnover rates among healthcare staff (8, 9).

Since compassion satisfaction and compassion fatigue are of great clinical importance, it is important to expand our knowledge of these constructs and capacity to measure their incidence, which cannot be achieved without psychometrically sound tools. On the top of that, it is hard to start treatment in the absence of screening and early detection. Furthermore, without valid and reliable tools, we cannot accurately measure the effectiveness of interventions aimed at increasing compassion satisfaction and reducing compassion fatigue. Therefore, the availability of sound instruments for measuring compassion fatigue and compassion satisfaction could help to provide better care for patients or clients in the future with earlier diagnosis of compassion fatigue, and therefore harm prevention.

## Measuring compassion fatigue and compassion satisfaction

Since compassion fatigue and compassion satisfaction have such a huge impact on the quality of care provided by helping professionals, it is striking that there are few valid and reliable tools to measure them. Additionally, the existing self-rated measuring tools have often been criticized over content and methodological issues, such as the lack of a total score for both positively and negatively worded items and for not being cross-culturally sensitive [e.g., Bride et al. (10) and Kristensen et al. (61)]. As Bride et al. (10) put it, “no single compassion fatigue measure assesses all aspects of the concept of compassion fatigue (i.e., trauma symptoms, cognitive distortions, general psychological distress, burnout, etc.).”

According to Bride et al. (10), the Compassion Fatigue Self-Test (CFST; 60) is the first instrument to measure compassion fatigue. The original version of the CFST consists of 40 items divided into two subscales: compassion fatigue and burnout. Stamm and Figley (11) later revised the CFST by adding questions to measure compassion satisfaction, which resulted in a 66-item version. Several attempts were made to shorten the over-long CFST and improve usage but another problem was that neither the factor structure of the test nor its psychometric properties were published and so remained unknown (10).

Gentry et al. (51) used the Compassion Fatigue Scale—Revised (CFS-R) with a shortened scale of 30 items for measuring compassion fatigue and burnout. Similarly, Adams et al. (63) developed the Compassion Fatigue Short Scale (CF-Short Scale) which has 13-items and two subscales—burnout and secondary trauma. The tool most commonly used to measure compassion fatigue and satisfaction is the Professional Quality of Life Scale (ProQOL; 52) which is a revised version of the Compassion Fatigue Self Test (60). It has three subscales: compassion satisfaction, burnout, and compassion fatigue/secondary traumatic stress. The ProQOL is comprised of 30-items. Since its creation in 1995, the ProQOL has been revised and updated several times. The latest version is the Professional Quality of Life Scale version 5 [ProQOL-5; Stamm (12)]. However, Bride, Radey and Figley (10) note that the validity and factor structure of the ProQOL have not been sufficiently studied. Most authors report only the reliability coefficients. Keesler and Fukui (13) reported that the original three-factor model was not a good fit with the data. Their solution was to delete 7 of the 30 items so that the factor analysis yielded satisfactory results for the three factors. Similarly, Duarte (14) and other authors (15, 16) reported difficulty fitting the factor structure of the ProQOL. Likewise, a meta-analysis by Hotchkiss and Wong (17) found problems with the factor structure of the ProQOL across 27 different cultures and languages. In summary, Hemsworth et al. (15) invited researchers to revise and improve ProQOL 5, while Wessels et al. (18) went even further, stating that there was widespread recognition that the lack of assessment instruments with good psychometric properties supported by rigorous research was seriously hindering both further research developments in the area and attempts to help practicing professionals. The existence of a reliable and valid measure of compassion fatigue and compassion satisfaction could provide important information for early screening, diagnosis, intervention, or treatment, and thereby ensure high-quality care for patients, clients, and customers.

### The research aim

Compassion fatigue and satisfaction significantly impact the quality of care provided by helping professionals, yet few valid and reliable tools exist to measure them. The Compassion Fatigue Self-Test (CFST; 60) was the first instrument to measure compassion fatigue. Despite several attempts to shorten and improve the CFST, its factor structure and psychometric properties remained unpublished and unknown (10).

The most frequently used version of the CFST, The Professional Quality of Life Scale (ProQOL; 52) showed to have factor structure problems across 27 cultures and languages based on the meta-analysis by Hotchkiss and Wong (17). Therefore, Wessels et al. (18) highlighted the lack of assessment instruments with robust psychometric properties as a major barrier to further research and practical applications.

To date, there is no scale that measures both compassion satisfaction and compassion fatigue together that has good psychometric properties and is not too long and cumbersome for data collection purposes. Thus, the aim of the present study was to revise, improve, and shorten the Compassion Fatigue and Satisfaction Self-Test for Helpers (11) using Mokken scale analysis for polytomous items (18, 19). In addition, we wanted to create norms for early screening of helping professionals followed by early intervention norms.

## Methods

### Research sample

The research sample was collected online through social media, contacts through professional associations and databases, personal contacts, and by asking participants to both compete the online questionnaire and forward the link to colleagues. We used REDCap (www.project-redcap.org) as the data-gathering tool. All participants signed an online written consent form.

There were 2,320 participants in total from various helping professions (participants could choose from one of the following: doctor, dentist, psychiatrist, nurse, paramedic, physiotherapist, hospital attendants, home nurse, social worker, psychologist, psychotherapist, coach, nun/monk, teacher, special educator, therapeutic pedagogue, educator, speech therapist, policemen, lawyer, doula, lactation consultant, human resources worker, volunteer, priest/pastor, radiologist, trainer, mentor, professional parent, pharmacist, and other). The research sample consisted of 1783 (76.9%) women, 527 (22.7%) men and 2 (0.1%) non-binary participants. Eight participants (0.3%) chose the option: I do not wish to say. Mean age was 41.74 with SD 11.62 ranging from 18 to 76 years.

### Research instruments

We used the Compassion Fatigue and Satisfaction Self-Test for Helpers [CFST; Stamm and Figley (11)]. The CFST is a self-rated 66-item instrument measuring three subscales, namely, Compassion Fatigue (23 items), Compassion Satisfaction (26 items) and Burnout (16 items). Items are scored from 0 (not at all) to 5 (very often) on a Likert-type scale. Compassion satisfaction represents the joy of helping others, e.g., “I find that I learn new things from those I care for.” Compassion fatigue refers to the cost of caring (2), e.g., “I am pre-occupied with more than one person I help.” Burnout is defined as the state of work-related exhaustion, e.g., “I have felt weak, tired, run down as a result of my work as a helper.” The subscale scores are calculated separately for each subscale. The Slovak version of the CFST was first translated, then back translated, and any discrepancies were discussed and resolved with an expert panel consisting of the co-authors.

Research studies using the CFST have reported good reliability coefficients. Figley (60) reported a Cronbach’s alpha ranging from 0.86 to 0.94, and Rudolph et al. (5) reported reliability coefficients of 0.87 for Compassion satisfaction, 0.87 for Compassion fatigue, and 0.90 for Burnout. Ortlepp and Friedman (20)‘s reliability coefficients were 0.84 for compassion fatigue, 0.83 for burnout, and 0.85 for compassion satisfaction, while Conrad and Kellar-Guenther (21) obtained the following reliability coefficients: 0.84 for compassion fatigue, 0.84 for burnout, and 0.86 for compassion satisfaction. Similarly, Steed and Bicknell (22) reported reliability coefficients of 0.87, 0.78, and 0.91 for compassion fatigue, burnout, and compassion satisfaction, respectively. The problem with the previous studies (23) is the small participant samples ranging from 67 (22) to 142 (53) and 363 (21). In addition the samples were highly specific, such as therapists working with perpetrators of sexual abuse (22), which means the results cannot be generalized to other kinds of helping professionals. In addition, there is as yet no published information about the factor structure of the CFST (10), probably because of statistical problems with long scales. The short scale items usually have larger factor loadings, obtained by factor analysis, than the long-scale items (24).

### Data analysis

In this paper, we used Mokken scale analysis (18, 19); Sijtsma and Molenaar, (58); to identify items with solid psychometric properties in the Compassion Fatigue and Satisfaction Self-Test for Helpers [CFST; Stamm and Figley (11)]. First, the items were assigned to subscales based on the theoretical framework of Stamm and Figley (11). Second, we performed an iterative Mokken scale analysis (18, 19); Sijtsma and Molenaar (58) on the subscales to check the assumptions of the Mokken double monotonicity model. Where the assumptions of the Mokken double monotonicity model were violated, we identified the items that did not fit the model and removed them until the final subscale provided satisfactory results.

### Mokken scale analysis

We performed the Mokken scale analysis separately for each scale. The Mokken model is a nonparametric item-response model, which has to meet the following assumptions (19); Sijtsma and Molenaar (58):

1. Unidimensionality: all the items in the subscale measure a single attribute that is quantified by means of a latent variable denoted Theta.
2. Monotonicity: As Theta increases, the probability of an item scoring a value increases or remains constant but cannot decrease—that means the more a respondent possesses the measured attribute, the more likely s/he is to obtain scores that are representative of responses typical of the higher attribute level.
3. Local independence: items measuring the attribute should correlate positively when respondents vary by Theta. This is implied by the fact that respondents with higher Theta scores are expected to have higher scores for each item than respondents with lower Theta scores, which means these scores should covary. If we remove this source of variation, this relationship between the items should disappear. Consequently, Theta should be the only source of variation and the items will be locally independent.

These three assumptions constitute the monotone homogeneity model for ordering persons. The double monotonicity model for ordering persons and items is stronger (in fact, this is a special feature of the monotone homogeneity model), and validity is only exhibited when these additional assumptions are met:

1. Invariant item ordering: the double monotonicity model implies the ordering of items by means of mean item scores. In other words, item ordering from easiest to hardest in terms of difficulty should be equal for different-ability (Theta) respondents. The double monotonicity model directly implies such an invariant item ordering.
2. Reliability: we will use the Molenaar–Sijtsma (MS) method (59) to estimate test-score reliability. The MS method assumes a stronger double monotonicity model. Its values must be close to 0.90, and over 0.70.

Two important caveats:

1. Sijtsma and van der Ark (19) point out that many researchers overlook the fact that the assumption (4)—invariant item ordering, the defining feature of the double monotonicity model—should be tested separately, and that it is not implied by the fulfilment of other properties (e g. strong scalability etc.).
2. Again, Sijtsma and van der Ark (19) argue that testing the invariant item ordering by means of the assumption that item step response functions (ISRF) should not intersect is inappropriate: a set of non-intersecting ISRFs does not directly imply an invariant item ordering.

### Procedure

All the analyses were performed in R version 4.2.1 (25), “mokken” package (26). The procedure was as follows:

1. Unidimensionality: The procedure suggested in Sijtsma and van der Ark (19) was used—for each subscale, we fitted the iteratively automated item selection procedure (AISP), option genetic algorithm (27) with increasing threshold, and observed whether the emergence of one subscale was confirmed.
2. Scalability: on completion of the test of unidimensionality, the initial subscales suggested by AISP with a threshold of 0.3 were selected. Subscales were considered satisfactory when Loevinger’s coefficient *H* ≥ 0.400, with 0.400 ≤ *H* < 0.500 indicating a medium strong scale, and *H* ≥ 0.500 indicating a strong scale. Items in each subscale were removed manually when *H* < 0.30 (28). Items were removed stepwise by first removing those with the most serious violations and then estimating the model again. This procedure was repeated until Loevinger’s coefficient H for the overall scale reached the value *H* = 0.400 (a medium strong scale) but considering standard error to ensure that the population value was not lower due to sampling error (e.g., a value of *H* = 0.405 with standard error 0.013 was not considered satisfactory). If this procedure failed to confirm the Mokken scale, the threshold (Loevinger’s coefficient H for the overall scale) was lowered to value *H* = 0.300 (a weak scale). If this second procedure failed to confirm the Mokken scale, the final conclusion was that the items were unscalable.
3. Local independence: the method of conditional associations proposed by Straat et al. (29) was used. Items flagged as locally non-independent were removed.
4. Monotonicity: the method suggested by Junker and Sijtsma (30) was used. Items with significant violations of monotonicity are inspected visually if the violation(s) are large enough to affect the monotonicity of the item response functions—especially in larger datasets, even significant violations of monotonicity in some item step response functions of an item could have a negligible effect on the overall item response function of this item. Items which did not pass the visual inspection were removed.
5. Invariant item ordering: the method proposed by Ligtvoet et al. (31) was used. Items violating the invariant item ordering were removed.
6. Reliability was calculated using MS rho reliability Molenaar–Sijtsma reliability coefficient and reported in the tables of results.
7. All descriptive statistics and Loevinger’s coefficients H (with standard errors) for remaining items and subscales were calculated and reported.
8. Norms (percentile rank norms, *z*-score norms, stanine boundaries) were calculated and reported, together with standard errors and 95% confidence intervals.

## Results

### Missing data analysis

A missing data analysis was conducted before assessing the subscales. The percentage of responses missing at least one answer was 11.94% (280 out of 2,346), with 15 missing patterns. To impute the missing data, a Bayesian framework was used (32), implemented in “mi” R package (33). As the items are polytomous, an ordered-categorical model with logit link was used. After imputations, the final sample size was 2,320.

### Outliers

**Figure 1:**
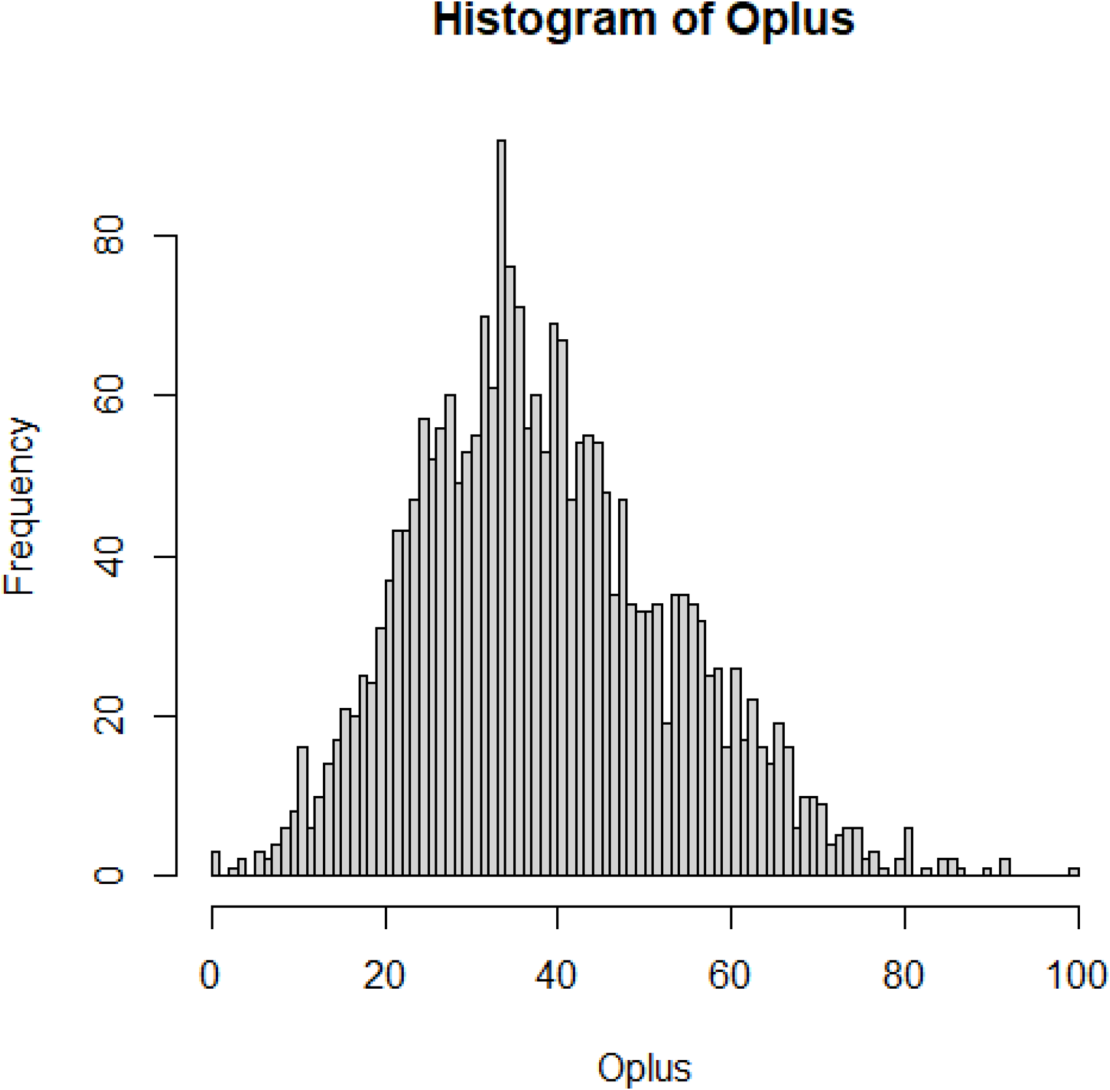
Distribution of errors for compassion fatigue.

**Figure 2:**
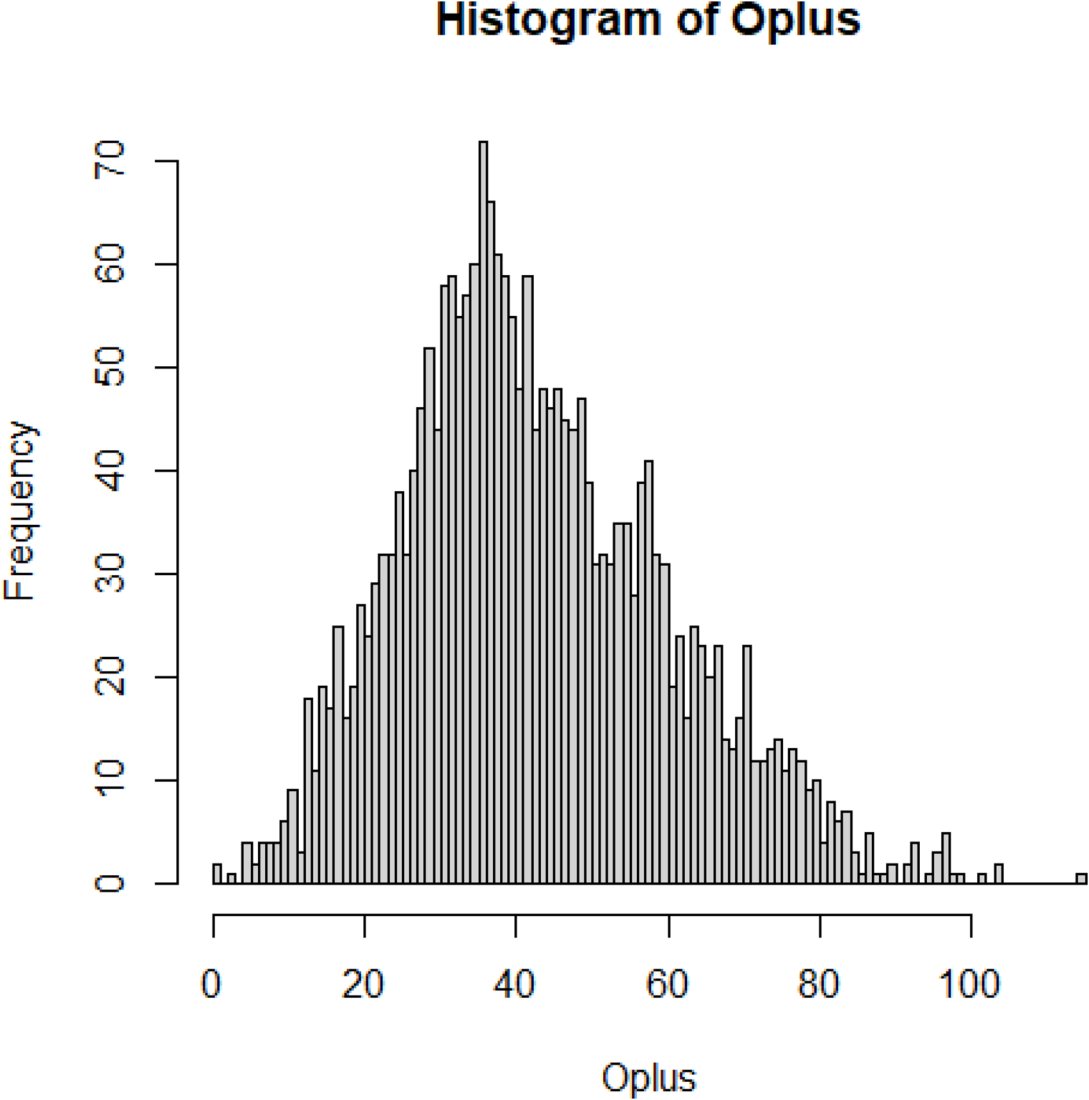
Distribution of errors for compassion satisfaction.

**Figure 3:**
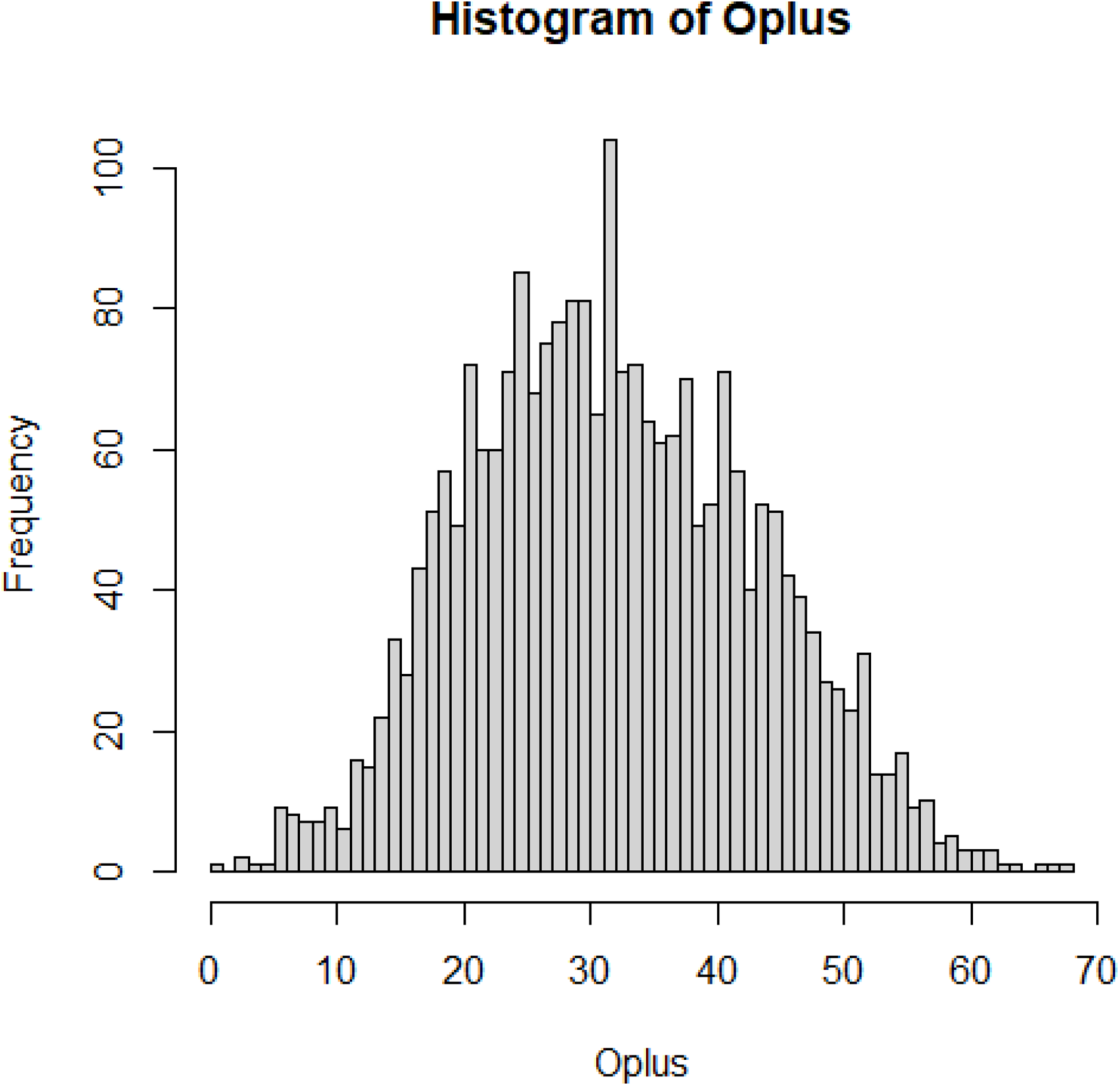
Distribution of errors for Burnout.

To handle outlying values for each subscale, the outlier detection method proposed by Zijlstra, Van der Ark and Sijtsma (34) was used. Given the relatively strong skew O_+_ distributions (Figures 1–3; Supplementary Appendix 1), we used adjusted boxplot (35) to accommodate the skewness. This produced a criterion value for the Compassion Fatigue subscale O_+_ = 10.53 (lower) and 93.00 (upper), and 30 outlying values were identified (1.28%), the criterion value for the Compassion Satisfaction subscale O_+_ = 10.67 (lower) and 110.26 (upper), and 28 outlying values were identified (1.19%), and a criterion value for the Burnout subscale O_+_ = 3.68 (lower) and 67.20 (upper), and only five outlying values were identified (0.21%). The analysis with and without the outliers shows a negligible influence on the outcomes (which comes as no surprise given their minimal rate).

### Unidimensionality of subscales

The automated item selection procedure (with genetic algorithm) failed to confirm a unidimensional scale for Compassion Satisfaction, but suggested two subscales. Therefore, we analyzed those subscales separately to check if they could constitute Mokken scales on their own.

### Descriptive analysis of the items

We calculated the descriptive statistics for items for all four subscales of the newly developed Compassion Satisfaction and Compassion Fatigue scale: Compassion Satisfaction Personal Integrity and Happiness, Compassion Satisfaction Work Competence and Happiness, Compassion Fatigue Burnout and Compassion Fatigue Secondary Traumatic Stress. See Tables 1–4. The descriptive statistics were mean, standard deviation, H item scalability coefficient, standard error of item scalability coefficient, and MS rho reliability Molenaar–Sijtsma reliability coefficient.

**Table 1:** Descriptive statistics of items of compassion fatigue secondary traumatic stress.

	Total	Total	Total	Total	Total	Total		
Items	0	1	2	3	4	5	M	Item H (SE)
Item 21	0.25	0.38	0.17	0.13	0.06	0.02	1.44	0.349 (0.016)
Item 28	0.40	0.33	0.12	0.11	0.03	0.01	1.09	0.418 (0.015)
Item 29	0.60	0.24	0.07	0.06	0.02	0.01	0.68	0.480 (0.014)
Item 31	0.37	0.34	0.13	0.10	0.04	0.02	1.14	0.444 (0.014)
Item 32	0.36	0.41	0.12	0.07	0.03	0.01	1.03	0.486 (0.013)
Item 34	0.57	0.27	0.08	0.05	0.02	0.01	0.71	0.470 (0.014)
Item 36	0.48	0.31	0.10	0.07	0.03	0.01	0.88	0.454 (0.014)
Item 39	0.37	0.30	0.15.	0.12	0.04	0.02	1.20	0.429 (0.014)
Item 40	0.34	0.32	0.17	0.11	0.05	0.02	1.27	0.436 (0.014)
MS rho	0.87							
Scale H (SE)	0.439 (0.011)							
Mean (SE)	9.434 (0.150)							
SD	7.257 (0.132)							
Range	0–43							

M, mean; SD, standard deviation; H, item scalability coefficient; SE, standard error of item scalability coefficient; MS rho, reliability Molenaar–Sijtsma reliability coefficient.

**Table 2:** Descriptive statistics of items of compassion fatigue Burnout.

	Total	Total	Total	Total	Total	Total		
Items	0	1	2	3	4	5	*M*	Item H (SE)
Item 41	0.35	0.34	0.15	0.11	0.03	0.01	1.17	0.450 (0.013)
Item 42	0.20	0.36	0.21	0.14	0.06	0.03	1.57	0.397 (0.014)
Item 48	0.06	0.25	0.27	0.26	0.12	0.04	2.26	0.416 (0.014)
Item 60	0.16	0.30	0.17	0.18	0.13	0.06	1.99	0.341 (0.015)
Item 62	0.28	0.36	0.16	0.14	0.04	0.02	1.37	0.486 (0.012)
Item 63	0.26	0.40	0.17	0.11	0.04	0.01	1.32	0.461 (0.013)
Item 64	0.19	0.36	0.20	0.15	0.07	0.03	1.61	0.441 (0.013)
MS rho	0.82							
Scale H (SE)	0.425 (0.011)							
Mean (SE)	11.286 (0.127)							
SD	6.175 (0.091)							
Range	0–35							

M, mean; SD, standard deviation; H, item scalability coefficient; SE, standard error of item scalability coefficient; MS rho, reliability Molenaar–Sijtsma reliability coefficient.

**Table 3:** Descriptive statistics of items of compassion satisfaction personal integrity and happiness.

	Total	Total	Total	Total	Total	Total		
Items	0	1	2	3	4	5	M	Item H (SE)
Item 1	0.01	0.04	0.09	0.20	0.48	0.19	3.68	0.597 (0.014)
Item 2	0.01	0.04	0.07	0.19	0.47	0.23	3.77	0.655 (0.013)
Item 3	0.01	0.03	0.06	0.12	0.42	0.36	4.00	0.540 (0.017)
Item 10	0.01	0.05	0.07	0.21	0.47	0.19	3.64	0.573 (0.014)
Item 14	0.02	0.06	0.11	0.26	0.43	0.12	3.39	0.581 (0.015)
MS rho	0.86							
Scale H (SE)	0.589 (0.012)							
Mean (SE)	18.473 (0.088)							
SD	4.260 (0.073)							
Range	0–25							

M, mean; SD, standard deviation; H, item scalability coefficient; SE, standard error of item scalability coefficient; MS rho, reliability Molenaar–Sijtsma reliability coefficient.

**Table 4:** Descriptive statistics of items of compassion satisfaction work competence and happiness.

	Total	Total	Total	Total	Total	Total		
Items	0	1	2	3	4	5	M	Item H (SE)
Item 30	0.01	0.04	0.12	0.21	0.47	0.15	3.54	0.398 (0.015)
Item 35	0.05	0.07	0.14	0.23	0.40	0.11	3.22	0.393 (0.015)
Item 47	0.01	0.05	0.11	0.23	0.44	0.16	3.53	0.394 (0.016)
Item 57	0.01	0.06	0.12	0.26	0.40	0.15	3.42	0.335 (0.016)
Item 66	0.02	0.05	0.08	0.13	0.42	0.30	3.75	0.386 (0.016)
MS rho	0.75							
Scale H (SE)	0.381 (0.013)							
Mean (SE)	17.462 (0.084)							
SD	4.072 (0.068)							
Range	0–25							

M, mean; SD, standard deviation; H, item scalability coefficient; SE, standard error of item scalability coefficient; MS rho, reliability Molenaar–Sijtsma reliability coefficient.

### Internal reliability

We calculated the internal reliability through Cronbach alpha polychoric coefficients. For the Compassion Fatigue and Satisfaction Self-Test for Helpers, the Cronbach’s *α* coefficient was 0.87 for Compassion Fatigue, 0.85 for Compassion Satisfaction, and 0.84 for Burnout subscales. For the Revised Compassion Satisfaction and Compassion Fatigue scale, reliability was calculated using MS rho (Molenaar–Sijtsma) reliability coefficient and indicated the following values for Compassion Satisfaction Personal Integrity and Happiness 0.86, Compassion Satisfaction Work Competence and Happiness 0.75, Compassion Fatigue Burnout 0.82 and Compassion Fatigue Secondary Traumatic Stress 0.87.

### Construct validity

The Mokken scale analysis helped us to shorten and improve the CSFT; the final version consisting of 26 items is given in Supplementary Appendix 1. The final item order of the Compassion Satisfaction and Compassion Fatigue scale was randomized via www.random.org.

### Norms

For the purposes of early diagnosis and consequently for immediate intervention or treatment and measuring the effectiveness of these, it is important to create a psychometrically sound scale as well as norms for the helping professional population. We calculated the Norms (percentile rank norms, z-score norms, stanine boundaries) and report them together with standard errors and 95% confidence intervals for each of the CSCFS subscales in Tables 5–16.

**Table 5:** *Z*-scores norms for compassion fatigue secondary traumatic stress.

Total	*Z*-scores SE	Lo	Up
0	−1.300 (0.020)	−1.338	−1.262
1	−1.162 (0.018)	−1.198	−1.126
2	−1.024 (0.017)	−1.058	−0.991
3	−0.887 (0.017)	−0.919	−0.854
4	−0.749 (0.016)	−0.781	−0.717
5	−0.611 (0.016)	−0.643	−0.579
6	−0.473 (0.017)	−0.506	−0.440
7	−0.335 (0.018)	−0.370	−0.301
8	−0.198 (0.019)	−0.234	−0.161
9	−0.060 (0.020)	−0.099	−0.021
10	0.078 (0.022)	0.036	0.120
11	0.216 (0.023)	0.170	0.261
12	0.354 (0.025)	0.304	0.403
13	0.491 (0.027)	0.438	0.545
14	0.629 (0.029)	0.572	0.686
15	0.767 (0.031)	0.706	0.828
16	0.905 (0.033)	0.839	0.970
17	1.043 (0.036)	0.973	1.113
18	1.180 (0.038)	1.106	1.255
19	1.318 (0.040)	1.240	1.397
20	1.456 (0.042)	1.373	1.539
21	1.594 (0.045)	1.506	1.682
22	1.732 (0.047)	1.639	1.824
23	1.870 (0.049)	1.773	1.966
24	2.007 (0.052)	1.906	2.109
25	2.145 (0.054)	2.039	2.251
26	2.283 (0.057)	2.172	2.394
27	2.421 (0.059)	2.305	2.536
28	2.559 (0.061)	2.438	2.679
29	2.696 (0.064)	2.571	2.822
30	2.834 (0.066)	2.704	2.964
31	2.972 (0.069)	2.837	3.107
32	3.110 (0.071)	2.970	3.249
33	3.248 (0.074)	3.103	3.392
34	3.385 (0.076)	3.236	3.534
35	3.523 (0.078)	3.369	3.677
36	3.661 (0.081)	3.502	3.820
38	3.937 (0.086)	3.768	4.105
39	4.074 (0.088)	3.901	4.247
42	4.488 (0.096)	4.300	4.675
43	4.626 (0.098)	4.433	4.818

SE, standard error; Lo, low bound; Up, upper bound.

**Table 6:** Stanines norms for compassion fatigue secondary traumatic stress.

Total	Stanines	SE	Lo	Up
1–2	−3.266	(0.181)	−3.621	−2.910
2–3	0.363	(0.138)	0.092	0.633
3–4	3.991	(0.118)	3.760	4.222
4–5	7.619	(0.132)	7.361	7.878
5–6	11.248	(0.172)	10.910	11.585
6–7	14.876	(0.225)	14.435	15.317
7–8	18.504	(0.283)	17.949	19.059
8–9	22.133	(0.344)	21.458	22.807

SE, standard error; Lo, low bound; Up, upper bound.

**Table 7:** Percentiles norms for compassion fatigue secondary traumatic stress.

Total	Percentiles	SE	Lo	Up
0	2.472	(0.224)	2.034	2.911
1	7.374	(0.489)	6.415	8.333
2	12.511	(0.639)	11.257	13.764
3	18.777	(0.758)	17.291	20.262
4	25.277	(0.862)	23.588	26.966
5	31.991	(0.920)	30.187	33.794
6	38.747	(0.974)	36.838	40.655
7	44.991	(0.993)	43.046	46.937
8	50.831	(1.005)	48.861	52.801
9	56.309	(0.993)	54.361	58.256
10	61.424	(0.981)	59.501	63.347
11	65.899	(0.954)	64.030	67.769
12	70.290	(0.919)	68.488	72.091
13	74.403	(0.877)	72.684	76.123
14	77.856	(0.839)	76.212	79.500
15	80.392	(0.806)	78.813	81.971
16	82.779	(0.761)	81.287	84.271
17	85.166	(0.718)	83.759	86.574
18	87.170	(0.676)	85.845	88.494
19	88.896	(0.636)	87.650	90.142
20	90.324	(0.599)	89.150	91.498
21	91.454	(0.568)	90.340	92.567
22	92.519	(0.532)	91.477	93.561
23	93.649	(0.492)	92.685	94.612
24	94.842	(0.441)	93.977	95.708
25	95.865	(0.401)	95.079	96.651
26	96.569	(0.367)	95.850	97.287
27	97.293	(0.322)	96.662	97.924
28	97.975	(0.280)	97.426	98.525
29	98.423	(0.250)	97.933	98.913
30	98.721	(0.226)	98.278	99.164
31	98.998	(0.198)	98.611	99.386
32	99.211	(0.179)	98.861	99.562
33	99.361	(0.159)	99.049	99.672
34	99.467	(0.149)	99.176	99.759
35	99.574	(0.128)	99.324	99.824
36	99.702	(0.109)	99.489	99.914
38	99.787	(0.090)	99.610	99.964
39	99.872	(0.067)	99.740100.004	
42	99.936	(0.048)	99.843100.029	
43	99.979	(0.021)	99.937100.020	

SE, standard error; Lo, low bound; Up, upper bound.

**Table 8:** *Z*-scores norms for compassion satisfaction personal integrity and happiness.

Total	*Z*-scores (SE)	Lo	Up
0	−4.336 (0.088)	−4.508	−4.164
2	−3.867 (0.080)	−4.023	−3.711
3	−3.632 (0.076)	−3.781	−3.483
4	−3.397 (0.072)	−3.538	−3.256
5	−3.163 (0.068)	−3.296	−3.029
6	−2.928 (0.064)	−3.053	−2.802
7	−2.693 (0.060)	−2.811	−2.575
8	−2.458 (0.056)	−2.569	−2.348
9	−2.224 (0.053)	−2.327	−2.121
10	−1.989 (0.049)	−2.084	−1.893
11	−1.754 (0.045)	−1.842	−1.666
12	−1.519 (0.041)	−1.600	−1.439
13	−1.285 (0.038)	−1.358	−1.211
14	−1.050 (0.034)	−1.117	−0.983
15	−0.815 (0.031)	−0.875	−0.755
16	−0.580 (0.027)	−0.634	−0.527
17	−0.346 (0.024)	−0.394	−0.298
18	−0.111 (0.022)	−0.154	−0.068
19	0.124 (0.020)	0.085	0.162
20	0.359 (0.018)	0.323	0.394
21	0.593 (0.017)	0.559	0.627
22	0.828 (0.017)	0.794	0.862
23	1.063 (0.019)	1.026	1.099
24	1.297 (0.020)	1.257	1.338
25	1.532 (0.023)	1.487	1.577

SE, standard error; Lo, low bound; Up, upper bound.

**Table 9:** Stanines norms for compassion satisfaction personal integrity and happiness.

Total	Stanines	SE	Lo	Up
1–2	11.018	(0.191)	10.643	11.392
2–3	13.148	(0.158)	12.838	13.457
3–4	15.278	(0.127)	15.029	15.526
4–5	17.408	(0.099)	17.213	17.602
5–6	19.538	(0.079)	19.382	19.693
6–7	21.668	(0.074)	21.523	21.812
7–8	23.798	(0.085)	23.630	23.965
8–9	25.928	(0.109)	25.715	26.141

SE, standard error; Lo, low bound; Up, upper bound.

**Table 10:** Percentiles norms for compassion satisfaction personal integrity and happiness.

Total	Percentiles	SE	Lo	Up
0	0.021	(0.021)	−0.020	0.063
2	0.085	(0.052)	−0.017	0.188
3	0.192	(0.082)	0.030	0.353
4	0.298	(0.109)	0.086	0.511
5	0.533	(0.136)	0.266	0.799
6	1.066	(0.194)	0.685	1.446
7	1.726	(0.256)	1.225	2.228
8	2.664	(0.312)	2.053	3.276
9	3.815	(0.381)	3.069	4.561
10	5.051	(0.435)	4.198	5.904
11	6.500	(0.493)	5.534	7.467
12	8.397	(0.551)	7.318	9.477
13	11.381	(0.625)	10.156	12.606
14	15.068	(0.711)	13.674	16.462
15	19.118	(0.783)	17.584	20.651
16	24.020	(0.849)	22.356	25.683
17	30.286	(0.908)	28.506	32.065
18	37.702	(0.959)	35.824	39.581
19	46.100	(0.981)	44.177	48.023
20	58.397	(0.933)	56.569	60.226
21	71.590	(0.867)	69.891	73.289
22	80.307	(0.777)	78.783	81.830
23	86.999	(0.640)	85.745	88.253
24	93.286	(0.453)	92.398	94.175
25	98.082	(0.198)	97.693	98.470

SE, standard error; Lo, low bound; Up, upper bound.

**Table 11:** *Z*-scores norms for compassion satisfaction work competence and happiness.

Total	*Z*-scores (SE)	Lo	Up
0	−4.288 (0.082)	−4.449	−4.128
2	−3.797 (0.074)	−3.942	−3.652
3	−3.552 (0.070)	−3.689	−3.414
4	−3.306 (0.066)	−3.436	−3.176
5	−3.060 (0.062)	−3.183	−2.938
6	−2.815 (0.058)	−2.929	−2.700
7	−2.569 (0.055)	−2.676	−2.462
8	−2.324 (0.051)	−2.423	−2.224
9	−2.078 (0.047)	−2.170	−1.986
10	−1.833 (0.043)	−1.917	−1.748
11	−1.587 (0.040)	−1.665	−1.509
12	−1.341 (0.036)	−1.412	−1.271
13	−1.096 (0.033)	−1.160	−1.032
14	−0.850 (0.029)	−0.908	−0.792
15	−0.605 (0.026)	−0.656	−0.553
16	−0.359 (0.024)	−0.406	−0.312
17	−0.113 (0.021)	−0.155	−0.071
18	0.132 (0.020)	0.093	0.171
19	0.378 (0.019)	0.341	0.415
20	0.623 (0.019)	0.586	0.660
21	0.869 (0.020)	0.830	0.908
22	1.115 (0.021)	1.073	1.156
23	1.360 (0.023)	1.314	1.406
24	1.606 (0.026)	1.555	1.657
25	1.851 (0.029)	1.794	1.908

SE, standard error; Lo, low bound; Up, upper bound.

**Table 12:** Stanines norms for compassion satisfaction work competence and happiness.

Total	Stanines	SE	Lo	Up
1–2	10.336	(0.171)	10.000	10.672
2–3	12.372	(0.142)	12.094	12.650
3–4	14.408	(0.115)	14.183	14.633
4–5	16.444	(0.092)	16.263	16.625
5–6	18.480	(0.079)	18.326	18.634
6–7	20.515	(0.078)	20.362	20.668
7–8	22.551	(0.091)	22.373	22.730
8–9	24.587	(0.113)	24.366	24.809

SE, standard error; Lo, low bound; Up, upper bound.

**Table 13:** Percentiles norms for compassion satisfaction work competence and happiness.

Total	Percentiles	SE	Lo	Up
0	0.064	(0.037)	−0.008	0.136
2	0.149	(0.077)	−0.001	0.300
3	0.213	(0.090)	0.036	0.390
4	0.405	(0.118)	0.173	0.637
5	0.725	(0.164)	0.402	1.047
6	1.023	(0.201)	0.629	1.417
7	1.471	(0.234)	1.011	1.930
8	2.323	(0.292)	1.750	2.896
9	3.367	(0.357)	2.667	4.068
10	5.030	(0.423)	4.200	5.859
11	7.374	(0.515)	6.364	8.385
12	10.209	(0.596)	9.040	11.378
13	13.853	(0.683)	12.515	15.191
14	18.457	(0.766)	16.956	19.957
15	24.808	(0.846)	23.151	26.466
16	32.694	(0.922)	30.887	34.501
17	40.665	(0.973)	38.758	42.572
18	49.808	(0.976)	47.895	51.721
19	60.678	(0.948)	58.820	62.536
20	72.187	(0.854)	70.513	73.860
21	81.650	(0.751)	80.178	83.121
22	87.873	(0.630)	86.639	89.107
23	93.009	(0.475)	92.079	93.940
24	96.697	(0.331)	96.048	97.345
25	98.977	(0.146)	98.691	99.263

SE, standard error; Lo, low bound; Up, upper bound.

**Table 14:** Z-scores norms for compassion fatigue Burnout.

Total	*Z*-scores (SE)	Lo	Up
0	−1.828 (0.027)	−1.880	−1.775
1	−1.666 (0.025)	−1.715	−1.616
2	−1.504 (0.024)	−1.550	−1.457
3	−1.342 (0.022)	−1.386	−1.298
4	−1.180 (0.021)	−1.222	−1.138
5	−1.018 (0.020)	−1.058	−0.978
6	−0.856 (0.020)	−0.895	−0.818
7	−0.694 (0.019)	−0.732	−0.657
8	−0.532 (0.019)	−0.570	−0.495
9	−0.370 (0.019)	−0.408	−0.333
10	−0.208 (0.020)	−0.247	−0.170
11	−0.046 (0.020)	−0.086	−0.006
12	0.116 (0.021)	0.074	0.157
13	0.277 (0.023)	0.233	0.322
14	0.439 (0.024)	0.393	0.486
15	0.601 (0.025)	0.552	0.651
16	0.763 (0.027)	0.710	0.816
17	0.925 (0.029)	0.869	0.982
18	1.087 (0.031)	1.027	1.147
19	1.249 (0.032)	1.185	1.313
20	1.411 (0.034)	1.344	1.479
21	1.573 (0.036)	1.502	1.644
22	1.735 (0.038)	1.660	1.810
23	1.897 (0.041)	1.817	1.976
24	2.059 (0.043)	1.975	2.142
25	2.221 (0.045)	2.133	2.309
26	2.383 (0.047)	2.291	2.475
27	2.545 (0.049)	2.448	2.641
28	2.707 (0.051)	2.606	2.807
29	2.869 (0.054)	2.764	2.974
30	3.030 (0.056)	2.921	3.140
31	3.192 (0.058)	3.079	3.306
32	3.354 (0.060)	3.236	3.472
33	3.516 (0.063)	3.394	3.639
34	3.678 (0.065)	3.551	3.805
35	3.840 (0.067)	3.709	3.972

SE, standard error; Lo, low bound; Up, upper bound.

**Table 15:** Stanines norms for compassion fatigue Burnout.

Total	Stanines	SE	Lo	Up
1–2	0.480	(0.161)	0.164	0.795
2–3	3.567	(0.134)	3.305	3.830
3–4	6.655	(0.119)	6.421	6.889
4–5	9.743	(0.121)	9.506	9.979
5–6	12.830	(0.138)	12.560	13.100
6–7	15.918	(0.166)	15.593	16.243
7–8	19.005	(0.201)	18.612	19.398
8–9	22.093	(0.239)	21.625	22.561

SE, standard error; Lo, low bound; Up, upper bound.

**Table 16:** Percentile norms for compassion fatigue Burnout.

Total	Percentiles	SE	Lo	Up
0	0.618	(0.114)	0.395	0.842
1	2.238	(0.268)	1.712	2.763
2	4.561	(0.397)	3.783	5.339
3	7.502	(0.511)	6.500	8.504
4	11.019	(0.614)	9.815	12.223
5	15.217	(0.708)	13.830	16.605
6	20.482	(0.794)	18.925	22.039
7	26.662	(0.875)	24.948	28.377
8	33.056	(0.936)	31.222	34.890
9	39.599	(0.974)	37.691	41.507
10	46.547	(0.992)	44.603	48.492
11	53.282	(0.997)	51.328	55.236
12	59.143	(0.986)	57.211	61.076
13	64.471	(0.959)	62.591	66.352
14	69.331	(0.927)	67.515	71.147
15	73.615	(0.886)	71.879	75.350
16	77.685	(0.834)	76.051	79.320
17	81.628	(0.774)	80.112	83.144
18	85.145	(0.711)	83.752	86.538
19	87.958	(0.652)	86.680	89.237
20	90.217	(0.595)	89.051	91.383
21	92.434	(0.523)	91.410	93.458
22	94.267	(0.465)	93.355	95.178
23	95.631	(0.404)	94.839	96.422
24	96.782	(0.351)	96.094	97.470
25	97.634	(0.300)	97.047	98.222
26	98.338	(0.252)	97.844	98.831
27	98.870	(0.206)	98.466	99.275
28	99.254	(0.168)	98.924	99.584
29	99.446	(0.150)	99.151	99.740
30	99.531	(0.138)	99.261	99.801
31	99.638	(0.118)	99.406	99.870
32	99.766	(0.093)	99.584	99.947
33	99.872	(0.067)	99.740100.004	
34	99.936	(0.048)	99.843100.029	
35	99.979	(0.021)	99.937100.020	

SE, standard error; Lo, low bound; Up, upper bound.

### Mokken analysis

Compassion Fatigue Secondary traumatic stress subscale.

Automated item selection procedure (with genetic algorithm) suggested a unidimensional scale with 12 items (20, 21, 22, 28, 29, 31, 32, 34, 36, 39, 40, 44) out of the original 23 items. Coefficient H for this subscale was *H* = 0.404 (0.010). Given the standard error, this value is not acceptable. After removing item 44 with *H* = 0.307 (0.016), the value for the subscale increased to *H* = 0.423 (0.010). Taking into account the standard error, this value is acceptable for a medium strong scale. Testing for local independence flagged item 20 as positively locally dependent with item 21. After removing item 20, the remaining 10 items were locally independent. The monotonicity test did not flag any of the items. The test for invariant item ordering flagged item 22. After removing item 22, the remaining items were locally independent, and the value for the subscale was *H* = 0.439 (0.011). The final set of items in this subscale was: 21, 28, 29, 31, 32, 34, 36, 39, 40 (medium strong scale). See Table 1 for details, and Supplementary Appendix 1 for the norms.

### Compassion fatigue burnout subscale

Automated item selection procedure (with genetic algorithm) suggested a unidimensional scale with 11 items (23, 24, 41, 42, 45, 48, 49, 60, 62, 63, 64) out of the original 17 items. Coefficient H for this subscale was *H* = 0.383 (0.009), which is not acceptable. After removing item 23 with *H* = 0.303 (0.014), the value for the subscale increased to *H* = 0.404 (0.009). Given the standard error, this value is still not acceptable. After removing another item (24) with *H* = 0.311 (0.015), the value for the subscale increased to *H* = 0.430 (0.010). Testing for local independence flagged item 49 as positively locally dependent with item 60, after removing it all the items were locally independent. The monotonicity test did not flag any of the items. The test for invariant item ordering flagged item 45. After removing item 45, all the remaining items were locally independent, and the value for the subscale was *H* = 0.439 (0.011). The final set of items in this subscale was: 41, 42, 48, 60, 62, 63, 64 (medium strong scale). See Table 3 for details, and Supplementary Appendix 1 for norms.

### Compassion satisfaction subscale

Automated item selection procedure (with genetic algorithm) failed to confirm a unidimensional scale, but suggested two subscales: the first subscale had 13 items (1, 2, 3, 9, 10, 14, 19, 26, 27, 46, 52, 53, 55) and the second subscale 6 items (30, 35, 47, 57, 61, 66). Therefore, we analyzed these subscales separately to see if they could constitute Mokken scales on their own.

### Compassion satisfaction personal integrity and happiness subscale

Testing for the local independence of the first subscale flagged 4 mutually positively locally dependent items (19, 27, 53, 55). After removing them, the remaining 9 items were locally independent. The monotonicity test did not flag any of the items. The test for invariant item ordering flagged items 9, 26, 46, 52. The final set of items in this subscale was 1, 2, 3, 10, 14, and the value for the subscale was *H* = 0.589 (0.012), a strong scale. See Table 2 for details, and Supplementary Appendix 1 for the norms.

### Compassion satisfaction work competence and happiness subscale

Testing for the local independence of the second subscale flagged one item (61) that was positively locally dependent with item 30. After removing it, all remaining 5 items were locally independent. The monotonicity test did not flag any items, and neither did the test for invariant item ordering. The final set of items in this subscale was 1, 2, 3, 10, 14, and the value for the subscale was *H* = 0.381 (0.013), a weak scale. See Table 2 for details, and Supplementary Appendix 1 for norms.

## Discussion

The aim of the present study was to revise the Compassion Fatigue and Satisfaction Self-Test for Helpers (11) using Mokken scale analysis for polytomous items (18, 19) to shorten it and improve its psychometric properties for diagnostic purposes. Additionally, we wanted to create norms for the helping professional population.

Contrary to previous research studies that used the CFST (11) with very small and highly specific samples of helping professionals [e.g., Figley (53) and Steed and Bicknell (22)], our research sample consisted of 2,320 participants from various helping professionals (more than 30 different helping professions) which allows us to generalize the results to all sorts of helping professionals and to create norms (percentile rank norms, z-score norms, and stanine boundaries) for the newly developed scale so that compassion fatigue and compassion satisfaction can be diagnosed among different kinds of helping professionals.

**Figure 4:**
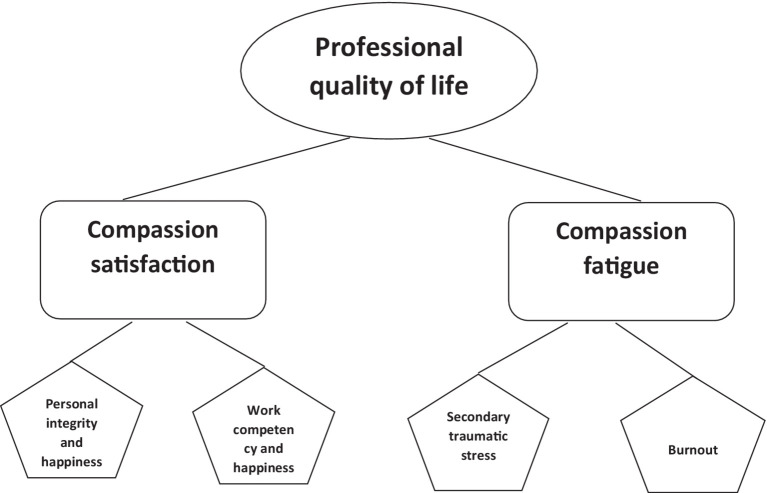
Model of professional quality of life.

Based on the Mokken scale analysis for polytomous items (18, 19), we deleted most of the items on the CFST (11) to improve scalability. The remaining items showed good scalability (with item scalability coefficients ranging from 0.349 to 0.655 and Molenaar–Sijtsma reliability coefficient between 0.75 and 0.87). As a result, we created a revised and shortened Compassion Satisfaction and Compassion Fatigue scale for the CFST (11) that has four subscales: Compassion Fatigue—Secondary Traumatic Stress, and Compassion Fatigue—Burnout were just shortened and renamed so as to better fit the proposed theory of Stamm (64); however, Compassion Satisfaction was divided into Compassion Satisfaction—Personal Integrity and Happiness, and Compassion Satisfaction—Work Competence and Happiness. The names of the subscales for the compassion fatigue items in our results correspond to the conceptualization that compassion fatigue is a combination of burnout and secondary traumatic stress (64). Accordingly, it corresponds to the names Adams et al. (63) attributed to the subscales of the shortened version of the CSFT (CF-Short Scale): burnout and secondary trauma. All the subscales of the newly developed scale are reliable and have high scalability. As a result, the final version of the revised and shortened CSFT consists of the following four subscales Compassion Satisfaction—Personal Integrity and Happiness (5 items) and Compassion Satisfaction—Work Competence and Happiness (5 items), Compassion fatigue—Secondary Traumatic Stress (9 items) and Compassion Fatigue—Burnout (7 items). The original CSFT has 66 items and the revised shortened version of CSFT has 26 items. The robust statistical analysis allows us to contribute new findings to the theory of professional quality of life developed by Stamm (64). Compassion fatigue stayed as it was, but compassion satisfaction emerged to consist of Personal Integrity and Happiness and Work Competence and Happiness. See Figure 4. Up until now, none of the elements of compassion satisfaction had been theorized or analyzed so this is the first step toward identifying compassion satisfaction and its constituent elements.

The newly adapted and shortened scale has good reliability coefficients measured by Cronbach alpha. Regarding reliability, all the Cronbach alpha polychoric coefficients were between 0.75 and 0.89. As they were above 0.70, we can conclude it has good reliability. [e.g., Streiner and Norman (36)]. Our results therefore support previous research findings that reported good reliability coefficients for the CFST (5, 20–22, 60).

In the revised version of the CSCFS, Compassion Satisfaction has two subscales: Personal Integrity and Happiness, and Work Competency and Happiness. Personal Integrity and Happiness is mainly composed of items that demonstrate happiness, satisfaction, and calmness as opposed to stress (1. I am happy. 2. I find my life satisfying. 10. I feel calm) and captured personal integrity (3. I have beliefs that sustain me. 14. I am the person I always wanted to be) indicating that the person lives their life according to their beliefs which may be related to the spiritual sphere (2).

Work Competency and Happiness is comprised of items relating to work happiness and feeling competent at one’s job (30. I have happy thoughts about those I help and how I could help them. 35. I have joyful feelings about how I can help the victims I work with. 47. I feel like I have the tools and resources that I need to do my work as a helper. 57. I am pleased with how I am able to keep up with helping technology). There is one item that describes being a long-term helper (66. I plan to be a helper for a long time).

Similarly to Stamm (64), the compassion fatigue in the revised version of the CSCFS has two subscales: Secondary Traumatic Stress, and Burnout. The Secondary Traumatic Stress subscale of the CSCFS consists of items relating to the symptoms of PTSD (DSM-5., 2013) such as: Intrusion (29. I experience troubling dreams similar to those I help. 31. I have experienced intrusive thoughts of times with especially difficult people I helped. 32. I have suddenly and involuntarily recalled a frightening experience while working with a person I helped), changes in cognition and mood (28. I am frightened of things a person I helped has said or done to me. 36. I think that I might have been “infected” by the traumatic stress of those I help), Altered Arousal and Reactivity (34. I am losing sleep over a person I help’s traumatic experiences) but not in Avoidance. None of the avoidance-related items, be that avoidance of people, things, or activities, was retained in the revised subscale. However, there appears to be a degree of avoidance in the following items: 39. I have felt trapped by my work as a helper and 40. I have a sense of hopelessness associated with working with those I help. This suggests that helping professionals might seek avoidance, but that in practice the nature of their work does not allow them to be avoidant, unless they are willing to quit their job.

One of the selected items of Compassion Fatigue—Secondary Traumatic Stress that remained after the Mokken scale analysis related to previous trauma experience in the person’s life (21. I have had first-hand experience with traumatic events in my childhood). In a systematic review, Bryce et al. (37) indicated that childhood trauma or adversity was indeed associated with the helping professions as a career choice. As Malach-Pines and Yafe-Yanai (38) explains, people are frequently driven to choose a career which corresponds with their childhood experiences, satisfies unmet needs from childhood, and achieves family aspirations. “People strive to actively master what they passively suffer” ((39), p. 55). Eighty percent of mental health professionals reported traumatic experience (56). The correlation between previous traumatic experience and risk of compassion fatigue has been analyzed [e.g., Boscarino et al. (40)].

It is possible that the trauma of clients and patients that helping professionals work with echoes their own unprocessed trauma. It would be interesting to focus on this more in future research and to find out whether helping professionals might be more prone to experiencing secondary traumatisation because they have previously experienced trauma themselves. Further research could also focus on the process of shared trauma [e.g., Tosone (41)], on the possible predictors of exposure-to-repeated-trauma, on posttraumatic growth, on compassion satisfaction trajectory or of exposure-to-repeated-trauma and on compassion fatigue and on mental health problems trajectory.

Based on DSM-5 (42), just as the Compassion Fatigue Secondary Traumatic Stress items are similar to the symptoms of PTSD, Compassion Fatigue Burnout consists of items similar to the symptoms of depression and anxiety. The CSCFS items relating to Anxiety symptoms [DSM-5; American Psychiatric Association (42)] are (41. I have felt “on edge” about various things and I attribute this to working with certain people I help. 48. I have felt weak, tired, run down as a result of my work as a helper.). The CSCFS items relating to Depressive symptoms [DSM-5; American Psychiatric Association (42)] are (48. I have felt weak, tired, run down as a result of my work as a helper. 62. I have a sense of worthlessness/disillusionment/resentment associated with my role as a helper. 63. I have thoughts that I am a “failure” as a helper. 64. I have thoughts that I am not succeeding at achieving my life goals.). However, none of these would be sufficient for a diagnosis of anxiety or depression disorder as the items do not meet the minimum requirements of 3 out of 6 for anxiety or 5 out of 9 for depression. Additionally, the subscale contains items relating to work-life balance and avoidance of helping (60. I find it difficult separating my personal life from my helper life. 42. I wish that I could avoid working with some people I help).

One could hypothesize that in order for helping professionals to help others, they must themselves have a satisfying personal and work life, otherwise they could end up exhausted and drained of resources. According to Kessler et al. (43), being exposed to trauma does not cause pathology. The pivotal things that decide whether a person stays mentally healthy or not are resilience and social support (44). Surprisingly, none of the CSCFS items relate directly to personal relationships or social support. One might argue that happiness is not possible without personal relationships and social support and, therefore, they are included in somewhat more generalized items like “I am happy.” or “I find my life satisfying.”

To manage levels of compassion fatigue and increase compassion satisfaction, it appears that people should build a good life for themselves from the very beginning of their helping profession career, and that this should be part of the curriculum for all helping professionals so they can avoid or prevent compassion fatigue.

## Implications

The new scale for measuring compassion fatigue and compassion satisfaction is psychometrically sound and contains a small number of items, facilitating early screening and detection of symptomatology. This enables swift intervention to prevent compassion fatigue and ensure high-quality services. Creating norms for the CSCFS will help professionals make meaningful comparisons against the general population and be vigilant about the various degrees of compassion fatigue screening.

Moreover, the scale can be used for regular monitoring, aiding in the identification of trends and patterns in compassion fatigue and satisfaction over time (64). This can inform the development of targeted interventions and support programs tailored to the specific needs of healthcare professionals (45).

The scale’s brevity and ease of use also make it suitable for integration into routine assessments in various healthcare settings, promoting a culture of mental health awareness and proactive care (46). Additionally, its cross-cultural applicability can enhance global research efforts, allowing for comparative studies and the development of universal strategies to address compassion fatigue and promote well-being among healthcare providers (16).

Using this scale can lead to better resource allocation by identifying departments or teams most at risk of compassion fatigue, thereby directing support and interventions where they are most needed (47). This could ultimately reduce turnover rates and improve job satisfaction, contributing to a more stable and effective workforce (48).

## Limitations and future directions

The primary limitation of our study is that the sample consists entirely of Slovak helping professionals, which may introduce cultural biases and limit the generalizability of our findings to other populations. Consequently, the revised version of the Compassion Satisfaction and Compassion Fatigue Scale (CSCFS) may reflect cultural nuances specific to the Slovak context. To address this limitation, future research should test the CSCFS in diverse cultural settings and professional environments to assess its usability and validate its factor structure across a broader range of samples (16). Expanding the study by including different different healthcare professions separately, such as nursing, psychology, and social work, could provide a more comprehensive understanding of the scale’s applicability into specific helping professions (45). Additionally, longitudinal studies would be beneficial to evaluate the stability of the scale over time and its sensitivity to changes in compassion fatigue and satisfaction following various interventions (64). This approach would help in refining the scale and enhancing its utility in diverse contexts.

## Conclusion

The Compassion Satisfaction and Compassion Fatigue Scale (CSCFS) appears to be a reliable and valid measure for assessing compassion fatigue and satisfaction, facilitating early screening and diagnosis. This tool provides a valuable means for researchers and practitioners to identify and address compassion fatigue, enabling timely interventions that can enhance the well-being of healthcare providers (49). By measuring the effectiveness of interventions and treatments, the CSCFS helps ensure high-quality care for patients, clients, and customers. As the scale is adopted in various cultural contexts and professional settings, it has the potential to become a standard tool for assessing compassion-related outcomes, thereby contributing to improved healthcare systems and provider well-being globally (46, 50).

## Data Availability

The raw data supporting the conclusions of this article will be made available by the authors, without undue reservation.
