# Supplementary material for: The development of the Compassion Satisfaction and Compassion Fatigue scale
Source: Front Public Health. 2024 Dec 5;12:1406467. doi: 10.3389/fpubh.2024.1406467 (PMC11655197; doi:10.3389/fpubh.2024.1406467)
Supplement: Supplementary file 1 [file Data_Sheet_1.docx]

**Appendix 1**

The revised CSFT with original items order from the original CSFT included

**The Revised and Shortened Compassion Satisfaction and Compassion Fatigue scale**

Adapted from Figley (1995) and from Stamm and Figley (1996)

Helping others puts you in direct contact with other people’s lives. As you probably have experienced, your compassion for those you help has both positive and negative aspects. This scale helps you estimate your compassion status, which includes your risk of burnout, compassion fatigue and compassion satisfaction with helping others. Consider each of the following characteristics about you and your current situation. **Write in the number that honestly reflects how frequently you experienced these characteristics in the last work week.**

| 0 | 1 | 2 | 3 | 4 | 5 |
| --- | --- | --- | --- | --- | --- |
| Never | Rarely | A Few Times | Somewhat Often | Often | Very Often |

1. (60.) I find it difficult separating my personal life from my helper life.
2. (36.) I think that I might have been "infected" by the traumatic stress of those I help.
3. (41.) I have felt "on edge" about various things and I attribute this to working with certain people I help.
4. (39.) I have felt trapped by my work as a helper.
5. (3.) I have beliefs that sustain me.
6. (21.) I have had first-hand experience with traumatic events in my childhood.
7. (63.) I have thoughts that I am a “failure” as a helper.
8. (34.) I am losing sleep over a person I help's traumatic experiences.
9. (66.) I plan to be a helper for a long time.
10. (32.) I have suddenly and involuntarily recalled a frightening experience while working with a person I helped.
11. (2.) I find my life satisfying.
12. (30.) I have happy thoughts about those I help and how I could help them.
13. (40.) I have a sense of hopelessness associated with working with those I help.
14. (10.) I feel calm.
15. (48.) I have felt weak, tired, run down as a result of my work as a helper.
16. (62.) I have a sense of worthlessness/disillusionment/resentment associated with my role as a helper.
17. (42.) I wish that I could avoid working with some people I help.
18. (29.) I experience troubling dreams similar to those I help.
19. (31.) I have experienced intrusive thoughts of times with especially difficult people I helped.
20. (14.) I am the person I always wanted to be.
21. (35.) I have joyful feelings about how I can help the victims I work with.
22. (64.) I have thoughts that I am not succeeding at achieving my life goals.
23. (47.) I feel like I have the tools and resources that I need to do my work as a helper.
24. (28.) I am frightened of things a person I helped has said or done to me.
25. (1.) I am happy.
26. (57.) I am pleased with how I am able to keep up with helping technology.

**Scoring:**

Calculate score by summing up the following numbers from the following items for the particular subscale:

**Compassion satisfaction:**

Personal integrity and happiness (5 items): 5, 11, 14, 20, 25.

Work competence and happiness (5 items): 9, 12, 21, 23, 26.

**Compassion fatigue:**

Secondary traumatic stress (9 items): 2, 4, 6, 8, 10, 13, 18, 19, 24.

Burnout (7 items): 1, 3, 7, 15, 16, 17, 22.

**Risk and potential cut-off score:**

Your potential for Compassion Satisfaction subscale Personal integrity and happiness:

23 and above = extremely high potential; 21-22 = high potential; 19-20 = good potential; 16-18 = modest potential; 15 or below = low potential.
**_________Your score for Compassion Satisfaction subscale Personal integrity and happiness**

Your potential for Compassion Satisfaction subscale Work competence and happiness:

22 and above = extremely high potential; 20-21 = high potential; 18-19 = good potential; 15-17 = modest potential; 14 or below = low potential.
**_________Your score for Compassion Satisfaction subscale Work competence and happiness**

Your risk for Compassion Fatigue subscale Secondary traumatic stress:

3 or less = extremely low risk, 4-6 = low risk; 7-10 = moderate risk; 11-15 = high risk; 16 or more = extremely high risk.

**_________Your score for Compassion Fatigue subscale Secondary traumatic stress**

Your risk for Compassion Fatigue subscale Burnout:

6 or less = extremely low risk, 7-9 = low risk; 10-12 = moderate risk; 13-17 = high risk; 18 or more = extremely high risk.

**_________Your score for Compassion Fatigue subscale Burnout**

**Appendix 2**

Original items selected from The Compassion Fatigue and Satisfaction Self-Test for Helpers (CFST; Stamm & Figley, 1996) for the new instrument The Compassion Satisfaction and Compassion Fatigue scale (CSCFS; Halamová et al., 2023)

**Compassion Satisfaction subscale 1 Personal integrity and happiness**

______1. I am happy.

______2. I find my life satisfying.

______3. I have beliefs that sustain me.

______10. I feel calm.

______14. I am the person I always wanted to be.

**Compassion Satisfaction subscale 2 Work competency and happiness**

______30. I have happy thoughts about those I help and how I could help them.

______35. I have joyful feelings about how I can help the victims I work with.

______47. I feel like I have the tools and resources that I need to do my work as a

helper.

____57. I am pleased with how I am able to keep up with helping technology.

____66. I plan to be a helper for a long time.

**Compassion Fatigue subscale 1 Secondary traumatic stress**

_____21. I have had first-hand experience with traumatic events in my childhood.

_____28. I am frightened of things a person I helped has said or done to me.

_____29. I experience troubling dreams similar to those I help.

_____31. I have experienced intrusive thoughts of times with especially difficult people I helped.

_____32. I have suddenly and involuntarily recalled a frightening experience while working with a person I helped.

_____34. I am losing sleep over a person I help’s traumatic experiences.

______36. I think that I might have been “infected” by the traumatic stress of those I help.

______39. I have felt trapped by my work as a helper.

______40. I have a sense of hopelessness associated with working with those I help.

**Compassion Fatigue subscale 2 Burnout**

______41. I have felt “on edge” about various things and I attribute this to working

with certain people I help.

_____42. I wish that I could avoid working with some people I help.

____48. I have felt weak, tired, run down as a result of my work as a helper.

____60. I find it difficult separating my personal life from my helper life.

____62. I have a sense of worthlessness/disillusionment/resentment associated with

my role as a helper.

____63. I have thoughts that I am a “failure” as a helper.

____64. I have thoughts that I am not succeeding at achieving my life goals.
